# Social Inequalities in Hypertension, Dyslipidemia, and Cardiovascular Events Among Adults with Type 2 Diabetes: A Cross-Sectional Study from Saudi Arabia

**DOI:** 10.3390/healthcare13131480

**Published:** 2025-06-20

**Authors:** Nurah Maziad Alamro, Abdulaziz Nasser Alahmari, Mohammed Ali Batais, Talal Khalid Alsaeed, Abdulhadi Abdulaziz Alsalhi

**Affiliations:** 1 Department of Family and Community Medicine, College of Medicine, King Saud University, Riyadh 11451, Saudi Arabia; nmalamro@ksu.edu.sa (N.M.A.); drmohammed34@gmail.com (M.A.B.); 2 Department of Family and Community Medicine, King Saud University Medical City, Riyadh 11451, Saudi Arabia; 3 Department of Family Medicine, King Saud University Medical City, Riyadh 11472, Saudi Arabia; 4 My Clinic International Medical Complex, Riyadh 13321, Saudi Arabia; 5 Department of Family Medicine, Ministry of Health, Riyadh, 11796, Saudi Arabia; t.k.alsaeed@hotmail.com; 6 Department of Family Medicine, Ministry of Interior, Riyadh 11134, Saudi Arabia; salhi762@gmail.com

**Keywords:** diabetes mellitus, type 2 diabetes, cardiometabolic risk, macrovascular complications, socioeconomic inequalities

## Abstract

**Background**: The present study seeks to examine how social disparities relate to the prevalence of poor glycemic control (HbA1c ≥ 7%), comorbidities such as hypertension and dyslipidemia, and diabetes-related complications (microvascular or macrovascular) among Saudi patients diagnosed with type 2 diabetes. **Methods**: A cross-sectional study was conducted among 574 patients with type 2 diabetes mellitus (T2DM) attending family medicine clinics at King Saud University Medical City in Riyadh. Participants were selected using a simple random sampling technique and interviewed via phone using a validated questionnaire. Data collected included demographic and clinical variables. Descriptive statistics and multivariate logistic regression analyses were performed to assess the association between socioeconomic status (SES) and cardiovascular complications, including stroke, dyslipidemia, hypertension, and acute coronary syndrome. **Result**: The analysis revealed that certain socioeconomic factors significantly increased the odds of cardiovascular complications among patients with T2DM. Being female was associated with higher odds of hypertension (OR = 2.29, *p* = 0.014), dyslipidemia (OR = 2.59, *p* = 0.012), acute coronary syndrome (ACS) (OR = 2.35, *p* = 0.001), and stroke (OR = 2.17, *p* = 0.003). Divorced or widowed participants had significantly increased odds of ACS (OR = 2.91, *p* = 0.001) and stroke (OR = 2.83, *p* = 0.002). A lower educational level (secondary school or less) was significantly associated with increased odds of hypertension (OR = 2.64, *p* = 0.031), dyslipidemia (OR = 2.22, *p* = 0.005), and stroke (OR = 2.88, *p* = 0.042). Monthly income between 3001 and 6000 SAR was significantly associated with higher odds of ACS (OR = 2.61, *p* = 0.003) and stroke (OR = 2.64, *p* = 0.012). Participants with diabetes duration >15 years had higher odds of dyslipidemia (OR = 2.86, *p* = 0.004) and stroke (OR = 2.89, *p* = 0.005). Being retired or not working increased the odds of all four cardiovascular outcomes, with stroke showing the highest risk (OR = 3.18, *p* < 0.001). Living outside the Riyadh region was also associated with elevated risk across outcomes, notably stroke (OR = 1.52, *p* = 0.046). **Conclusions**: The study concluded that notable social disparities exist among diabetic individuals affected by cardiovascular conditions, such as stroke and acute coronary syndrome (ACS), as well as risk factors for cardiovascular disease like dyslipidemia (DLD). These findings can inform targeted cardiovascular risk reduction strategies and address health inequities among diabetic populations in Saudi Arabia.

## 1. Introduction

Diabetes mellitus (DM) remains a significant global public health concern [1]. The World Health Organization (WHO) defines DM as “a chronic, metabolic disease characterized by elevated levels of blood glucose (or blood sugar), which leads over time to serious damage to the heart, blood vessels, eyes, kidneys and nerves” [2,3,4,5].

Type 2 diabetes mellitus (T2DM), formerly referred to as “non-insulin-dependent” or “adult-onset” diabetes, results from the body’s inability to use insulin effectively [6]. It accounts for the majority of diabetes cases worldwide and is primarily associated with excess body weight, including overweight and obesity, as well as insufficient physical activity [6]. In the United States, diabetes is widespread. According to CDC data from 2020, about 34.2 million Americans—just over 10% of the population—have diabetes, while roughly 88 million (or one in three) are affected by prediabetes. Globally, diabetes affects people across all regions, including low- and middle-income countries [7]. Without effective preventive interventions, the global number of individuals with diabetes is projected to reach approximately 630 million by 2045 [8]. Each year, elevated blood sugar levels are responsible for over 4 million deaths [9]. Individuals with diabetes are particularly susceptible to developing other serious health issues, especially cardiovascular diseases [10]. Cardiovascular disease encompasses various conditions, such as coronary heart disease (CHD), cardiomyopathy, arrhythmias, and heart failure [11].

Evidence from research indicates that the risk of developing cardiovascular diseases increases with the duration of diabetes. Cardiovascular disease encompasses various conditions, such as coronary heart disease (CHD), cardiomyopathy, arrhythmias, and heart failure. CHD is defined as the accumulation of cholesterol-rich plaque within the coronary arteries [12]. This plaque buildup significantly elevates the likelihood of heart attacks among individuals with diabetes. Furthermore, diabetic patients are at increased risk of hypertension, as the heart must exert more force to circulate blood throughout the body [13]. Elevated blood glucose levels cause red blood cells (RBCs) to adhere to the inner linings of blood vessels. As more RBCs accumulate, they begin to cluster and obstruct normal blood flow [14]. This narrowing of the vessels results in increased blood pressure, forcing the heart to work harder to supply blood to various organs. The repeated impact of blood cells against the vessel walls contributes to vascular damage [13].

Impaired blood flow can prevent vital organs like the heart from receiving sufficient oxygen and nutrients, potentially leading to cardiovascular complications. Dyslipidemia (DLD) refers to abnormal lipid profiles in the blood, including elevated cholesterol or triglyceride levels, or reduced high-density lipoprotein (HDL) levels, all of which can contribute to atherosclerosis [9,10,11]. The combination of excess cholesterol and fats as well as metabolic disturbances plays a critical role in the development of cardiovascular conditions [15]. Advancements in technology and improvements in living standards have played a major role in enhancing health outcomes [16]. However, these improvements are not experienced equally across populations. Health disparities are largely shaped by the social and environmental conditions in which individuals are born and raised, including factors such as income level and educational attainment [17]. While various cardiovascular complications are frequently observed among patients with T2DM, their prevalence still varies due to several contributing factors [18]. Socioeconomic disparities have been identified as influential in determining the risk of cardiovascular issues among individuals with type 2 diabetes [19]. This study aimed to examine the association between social disparities and the prevalence of hypertension, dyslipidemia, and cardiovascular complications among Saudi patients with type 2 diabetes mellitus.

## 2. Methodology

### 2.1. Study Design and Participants

This cross-sectional study was carried out between February 2020 and May 2021 among individuals diagnosed with type 2 diabetes mellitus (T2DM) who were receiving care at the family medicine clinics of King Saud University Medical City in Riyadh. A total of 574 participants were included in the sample, which was calculated using a single proportion formula with an additional 10% added to account for potential non-responses.

### 2.2. Data Collection

The family medicine clinics maintain an electronic database of all patients under regular follow-up. From this database, patients who met the inclusion criteria—diagnosed with type 2 diabetes for over one year and aged 18 years or older—were identified. Using a simple random sampling method, 574 eligible patients were selected for participation in the study. No explicit exclusion criteria were applied for this study, other than the requirement that participants be adults (≥18 years) with a confirmed diagnosis of type 2 diabetes mellitus.

Given the constraints imposed by the COVID-19 pandemic, data collection was conducted through telephone interviews. Each participant was contacted using a designated phone line. At the start of every call, an oral consent form was read to the patient, outlining the study’s purpose, objectives, expected participation time, potential benefits, contact information for further inquiries, and a statement affirming the participant’s right to withdraw at any stage. Verbal consent was then obtained before proceeding.

Although several instruments assess diabetes-related comorbidities and social determinants—such as the WHO STEPS survey for non-communicable diseases and the Diabetes Self-Management Questionnaire (DSMQ)—we adopted the tool developed by Petrie et al. [13] for three main reasons. The first involves scope and content; the questionnaire captures both clinical complications (e.g., stroke, ACS, dyslipidemia) and key socioeconomic indicators, aligning precisely with our study aims. The second reason involves prior validation in a Saudi population; its original Arabic version demonstrated excellent internal consistency (Cronbach’s α = 0.82) and test–retest reliability, minimizing the cultural-translation bias that can occur with internationally developed tools. The third involves feasibility for telephone administration during the COVID-19 pandemic; the instrument contains primarily closed-ended items, allowing completion in approximately 12 min without compromising data quality. Collectively, these attributes made it the most suitable choice over alternative questionnaires for our target setting and research objectives. The questionnaire had been professionally translated into Arabic by Hussain Alrikabi, an accredited translator certified by the National Accreditation Authority for Translators and Interpreters (NAATI). It included items addressing demographic information as well as detailed inquiries into the participants’ medical histories. Data on comorbid conditions were gathered both using patient self-reports and by reviewing their medical records.

The study variables included (1) demographic characteristics; (2) cardiovascular morbidities, identified either with self-reported history or confirmed diagnoses of conditions such as stroke, coronary artery disease, history of coronary procedures, lower limb ulcers, or limb amputations; (3) diabetic retinopathy, defined by an ophthalmologist-confirmed diagnosis or documented clinical records; (4) neuropathy, indicated either by clinical diagnosis of peripheral neuropathy or self-reported symptoms including numbness or tingling pain in the lower extremities. Diabetic nephropathy was defined based on eGFR < 60 mL/min/1.73 m^2^, acknowledging that the absence of albuminuria data may have led to an underestimation of early kidney damage [6,7,8].

Glycemic control was assessed using the most recent HbA1c values retrieved from patient records. Glycemic status was categorized as controlled (<53 mmol/mol or <7%), partially controlled (53–63 mmol/mol or 7% to <8%), and poorly controlled (≥64 mmol/mol or ≥8%) [20]. An additional category, very poorly controlled diabetes, was defined as HbA1c > 68 mmol/mol (≥9%) [21]. Fasting blood glucose values above 130 mg/dL were also used to define poor glycemic control [22].

Lipid profile abnormalities were defined as follows: LDL levels > 2.0 mmol/L (77.3 mg/dL), triglycerides > 2.0 mmol/L (177.1 mg/dL), and HDL levels < 1.0 mmol/L (38.7 mg/dL) [23]. Hypertension was classified based on documented diagnosis or the use of antihypertensive medication, or if the average of the two most recent blood pressure readings exceeded 140 mmHg systolic or 90 mmHg diastolic [24,25].

Microalbuminuria was defined as an albumin-to-creatinine ratio (ACR) of 30–300 mg/g, while macroalbuminuria was indicated by an ACR exceeding 300 mg/g [26]. Body mass index (BMI) was categorized according to World Health Organization standards as follows: normal (<25.0 kg/m^2^), pre-obese (25.0–29.9 kg/m^2^), and obese (≥30.0 kg/m^2^, encompassing class I, II, and III obesity) [27]. While the dataset has been used in prior research, the current study explores different outcome variables and hypotheses related to cardiovascular risk.

### 2.3. Statistical Analyses

Data analysis was conducted using JMP software (version 16.0, JMP business unit of the SAS Institute, Cary, NC, USA). Descriptive statistics were utilized to summarize the baseline characteristics of the study participants. To evaluate the relationship between socioeconomic factors and the occurrence of type 2 diabetes complications, multivariate logistic regression analyses were applied. In the first model, adjustments were made for the age variable alone, and the model included an assessment of general risk factors such as gender, marital status, educational attainment, monthly income, recent blood glucose levels, diabetes duration, employment status, and region of residence. The second model expanded the adjustment to encompass all these sociodemographic and clinical risk factors. Finally, the third model incorporated diabetes duration and the most recent fasting blood glucose level (mg/dL or mmol/L) as mediating variables to explore their potential role in the causal pathway between socioeconomic indicators and T2DM-related complications.

## 3. Result

### 3.1. Sociodemographic Characteristics

The sociodemographic details of the study participants are summarized in Table 1. The average age of participants was 57.93 years (±10.20). The study included an equal distribution of male and female participants (n = 287 each). Regarding marital status, the majority of participants were married (80.1%, n = 460), followed by widowed individuals (12.7%, n = 73). Divorced participants made up 5.4% (n = 31), while those who were single constituted the smallest group (1.7%, n = 10).

In terms of educational attainment, 29.6% (n = 170) of the participants held a university degree. Illiterate individuals accounted for 20.4% (n = 117), those who had completed tertiary school comprised 19.9% (n = 114), and primary school graduates represented 17.4% (n = 100). The smallest educational group was intermediate school graduates, making up 12.7% (n = 73) of the sample.

The majority of the study population were Saudi nationals (93.2%, n = 535), while non-Saudi participants represented 6.8% (n = 39). Regarding employment status, homemakers and housewives formed the largest subgroup (36.9%, n = 212), followed by retirees (31.4%, n = 180). Participants who were currently employed made up 27.9% (n = 160). The smallest subgroups included those not working but capable of employment (2.1%, n = 12) and those unable to work (1.7%, n = 10).

The findings further indicated that 28% of participants reported a monthly income exceeding 12,000 SAR. Additionally, around 25.85% (n = 148) earned less than 3000 SAR per month. Another 21.4% (n = 123) reported a monthly income between 3001 and 6000 SAR, while 15.9% (n = 91) fell into the 6001–9000 SAR income bracket. The least represented income group comprised those earning between 9001 and 12,000 SAR, accounting for 8.9% (n = 51) of the sample.

In terms of housing, most participants (71.1%, n = 481) lived in villas, while 14.1% (n = 81) resided on a floor within a villa or multi-unit building. The average number of housing units was 2.82 (±0.97).

The mean duration of diabetes among the participants was 14.74 years (±8.9). More than half of the patients (58.5%, n = 336) reported receiving follow-up care for diabetes every three months, while 32.4% (n = 186) had follow-ups every six months. The majority (82.8%, n = 475) received care in public hospitals, and 13.8% (n = 79) attended specialized diabetes centers.

Regarding treatment modalities, 52.6% (n = 302) of participants used a combination of oral medications and insulin. Oral medications alone were used by 40.2% (n = 231), insulin alone by 6.3% (n = 36), and dietary management alone was reported by 0.9% (n = 5).

### 3.2. Cardiovascular Morbidities

Table 2 presents the odds ratios (ORs) for hypertension in relation to various socioeconomic status (SES) variables. In Model 1, which controlled for age, the results suggested that the odds of having hypertension tended to be higher among females (OR = 2.13, 95% CI: 0.61–2.33), married individuals (OR = 1.88, 95% CI: 0.66–2.44), and those with an education level of secondary school or below (OR = 2.07, 95% CI: 0.12–3.03), although these associations were not statistically significant because the confidence intervals included 1. Similarly, the odds for participants with a monthly income ranging from 3001 to 6000 SAR (OR = 2.25, 95% CI: 0.78–3.93), individuals with a diabetes duration of 6 to 15 years (OR = 2.41, 95% CI: 0.63–3.67), and those residing outside Riyadh (OR = 2.91, 95% CI: 2.54–3.48) also appeared to be elevated, but these findings were not statistically significant in most cases.

In Model 2, which adjusted for all general sociodemographic risk factors, similar patterns were observed. The odds of hypertension tended to be higher among women (OR = 2.13, 95% CI: 0.51–2.21), married participants (OR = 2.11, 95% CI: 0.97–2.26), and those with secondary education or less (OR = 2.57, 95% CI: 1.18–3.26). There were also elevated odds for participants with a monthly income between 3001 and 6000 SAR (OR = 2.18, 95% CI: 1.16–4.83), those who had lived with diabetes for 6 to 15 years (OR = 2.33, 95% CI: 1.03–3.84), and individuals not in active employment or retired (OR = 2.91, 95% CI: 2.21–6.14). The odds for participants residing outside Riyadh appeared to be higher (OR = 2.04, 95% CI: 0.30–3.51), but this association was not statistically significant. Similarly, the odds for participants with secondary education or less (OR = 2.64, 95% CI: 0.67–4.24) suggested an elevated risk, but the confidence interval included 1, indicating no strong evidence of a true association.

In Model 3, which included intermediate variables such as diabetes duration and recent fasting blood sugar levels, females remained at an elevated risk for hypertension (OR = 2.29, 95% CI: 0.57–2.34). However, the confidence interval included 1, so this was not statistically significant. Higher odds were observed among divorced or widowed individuals (OR = 2.36, 95% CI: 2.07–4.22), those with a secondary education or less (OR = 2.64, 95% CI: 0.67–4.24), participants with an income exceeding 6000 SAR (OR = 2.49, 95% CI: 0.81–3.29), and those living outside of Riyadh (OR = 2.17, 95% CI: 0.37–2.88). Although some subgroups showed higher odds ratios, many of these associations were not statistically significant due to confidence intervals including 1.

### 3.3. Dyslipidemia

Table 3 presents the odds ratios (ORs) for dyslipidemia (DLD) in relation to socioeconomic status (SES) variables. In Model 1, which was adjusted for age, the odds of DLD were significantly higher among female participants (OR = 2.16, 95% CI: 1.17–3.21) and married individuals (OR = 2.49, 95% CI: 1.18–3.89). The odds also appeared to be elevated among those holding a university degree (OR = 1.23, 95% CI: 0.26–1.58), patients with a monthly income between 3001 and 6000 SAR (OR = 2.09, 95% CI: 2.51–3.48), those living with diabetes for more than 15 years (OR = 2.11, 95% CI: 0.33–2.64), individuals who were retired or unemployed (OR = 2.33, 95% CI: 1.28–2.57), and those residing outside Riyadh (OR = 1.32, 95% CI: 0.55–2.25). However, many of these associations were not statistically significant, as their confidence intervals included 1.

In Model 2, which included adjustments for a broader set of sociodemographic variables, similar patterns were observed. The odds of DLD tended to be higher among females (OR = 2.32, 95% CI: 0.49–2.37) and divorced or widowed participants (OR = 2.74, 95% CI: 1.33–2.97). Elevated odds were also noted among those with a secondary school education or less (OR = 2.08, 95% CI: 2.20–3.15), patients earning between 3001 and 6000 SAR per month (OR = 2.09, 95% CI: 2.51–3.48), those with diabetes duration of more than 15 years (OR = 2.11, 95% CI: 0.33–2.64), participants who were retired or not employed (OR = 2.49, 95% CI: 1.61–3.52), and those living outside of Riyadh (OR = 1.41, 95% CI: 0.93–2.37). While some subgroups showed elevated odds, not all of these findings reached statistical significance.

In Model 3, which incorporated intermediate variables such as diabetes duration and recent blood glucose levels, the odds of DLD were significantly higher among females (OR = 2.59, 95% CI: 1.18–2.87) and married individuals (OR = 3.01, 95% CI: 1.87–3.08). Participants with a secondary school education or less (OR = 2.22, 95% CI: 2.01–3.33), those earning more than 6000 SAR monthly (OR = 2.59, 95% CI: 1.63–2.70), patients with diabetes for over 15 years (OR = 2.86, 95% CI: 1.81–4.63), individuals who were retired or unemployed (OR = 2.96, 95% CI: 2.29–4.33), and residents outside Riyadh (OR = 2.18, 95% CI: 0.31–2.51) also tended to have higher odds of dyslipidemia. However, some of these associations were not statistically significant because their confidence intervals included 1.

### 3.4. Acute Coronary Syndrome

Table 4 outlines the odds ratios (ORs) for acute coronary syndrome (ACS) in relation to socioeconomic status (SES) variables. In Model 1, which accounted for age, the odds of ACS tended to be higher among female participants (OR = 1.77, 95% CI: 0.67–2.41), those who were divorced or widowed (OR = 2.09, 95% CI: 1.41–2.56), and individuals with a secondary school education or lower (OR = 1.88, 95% CI: 0.33–2.57). Elevated odds were also noted among those earning more than 6000 SAR monthly (OR = 2.27, 95% CI: 1.15–3.62), patients with diabetes duration between 6 and 15 years (OR = 1.99, 95% CI: 0.86–3.12), retired or unemployed participants (OR = 1.96, 95% CI: 1.31–2.17), and those residing outside Riyadh (OR = 1.37, 95% CI: 0.38–1.52). However, some of these associations were not statistically significant, as their confidence intervals included 1.

In Model 2, which adjusted for a broader range of sociodemographic risk factors, the odds of having ACS remained higher among females (OR = 2.07, 95% CI: 1.65–2.12), divorced or widowed individuals (OR = 2.23, 95% CI: 1.79–3.16), and those with a secondary school-level education or below (OR = 2.16, 95% CI: 0.58–2.54). Elevated odds were also observed among patients with an income greater than 6000 SAR (OR = 2.41, 95% CI: 1.08–2.87), those with diabetes for more than 6 but fewer than 15 years (OR = 2.11, 95% CI: 1.84–2.53), retired or unemployed individuals (OR = 2.26, 95% CI: 1.49–3.51), and patients living outside Riyadh (OR = 1.40, 95% CI: 0.89–2.23). Many of these associations, however, were not statistically significant because their confidence intervals included 1.

Model 3, which included intermediate variables such as diabetes duration and fasting glucose levels, suggested the highest odds of ACS among females (OR = 2.35, 95% CI: 2.13–3.01) and divorced or widowed individuals (OR = 2.91, 95% CI: 1.62–4.07). Interestingly, patients with university-level education also tended to have higher odds (OR = 2.64, 95% CI: 2.00–3.67), as did those earning between 3001 and 6000 SAR monthly (OR = 2.61, 95% CI: 2.51–4.16), individuals with diabetes duration between 6 and 15 years (OR = 2.38, 95% CI: 2.17–3.43), those retired or unemployed (OR = 2.79, 95% CI: 2.36–3.17), and those residing outside Riyadh (OR = 1.66, 95% CI: 1.03–3.14). While higher odds ratios were noted for several subgroups, some of these associations were not statistically significant because their confidence intervals included 1.

### 3.5. Stroke

Table 5 presents the odds ratios (ORs) for stroke in relation to socioeconomic status (SES) indicators. In Model 1, which adjusted for age, the odds of stroke were higher among female participants (OR = 1.75, 95% CI: 1.13–2.56), divorced or widowed individuals (OR = 2.51, 95% CI: 1.51–2.70), and those with a secondary school education or lower (OR = 2.19, 95% CI: 1.05–2.89). Additionally, participants earning between 3001 and 6000 SAR monthly (OR = 1.84, 95% CI: 0.98–3.16), those with diabetes for over 15 years (OR = 2.17, 95% CI: 1.29–3.12), individuals who were retired or not working (OR = 2.61, 95% CI: 1.42–2.88), and residents outside Riyadh (OR = 1.35, 95% CI: 1.11–3.48) tended to have elevated odds, although some of these associations were not statistically significant as the confidence intervals included 1.

In Model 2, after adjusting for a broader range of sociodemographic variables, higher odds of stroke persisted among females (OR = 1.83, 95% CI: 0.27–2.68), divorced or widowed participants (OR = 2.69, 95% CI: 2.41–3.55), and those with a secondary school education or less (OR = 2.36, 95% CI: 2.07–3.61). Elevated odds were also noted for participants earning more than 6000 SAR monthly (OR = 2.24, 95% CI: 2.18–3.55), those with diabetes for over 15 years (OR = 2.39, 95% CI: 1.18–2.69), those not employed or retired (OR = 2.83, 95% CI: 1.27–5.12), and individuals residing outside Riyadh (OR = 1.34, 95% CI: 0.58–2.69). While some of these odds ratios were elevated, not all were statistically significant, as indicated by confidence intervals including 1.

In Model 3, which incorporated intermediate variables such as diabetes duration and fasting glucose levels, the odds of stroke remained higher among females (OR = 2.17, 95% CI: 2.12–2.59), divorced or widowed participants (OR = 2.83, 95% CI: 2.30–3.57), and those with secondary education or less (OR = 2.88, 95% CI: 1.01–5.15). Higher odds were also observed for participants earning between 3001 and 6000 SAR (OR = 2.64, 95% CI: 1.69–3.08), those with more than 15 years of diabetes (OR = 2.89, 95% CI: 1.17–5.66), individuals retired or not working (OR = 3.18, 95% CI: 2.94–3.67), and participants living outside Riyadh (OR = 1.52, 95% CI: 0.81–3.00). Although some subgroups demonstrated elevated odds ratios, many of these associations were not statistically significant because their confidence intervals included 1.

## 4. Discussion

Health equity within any healthcare system is shaped by several critical contextual determinants. The first is economic stability, encompassing factors such as employment status, income level, financial obligations, medical expenses, and access to financial support. The second is the physical environment, which includes aspects like housing conditions, transportation availability, and personal safety. A third determinant is education, which covers literacy, early education, vocational training, and access to higher education opportunities [28]. The fourth factor is food access, particularly food security and the availability of healthy dietary options. The fifth is community and social context, involving elements such as social integration, support networks, community involvement, psychosocial stress, and exposure to violence. Lastly, the healthcare system itself plays a key role, including dimensions such as access to health coverage, provider availability, culturally and linguistically competent care, and overall quality of services [29]. For example, research by Pérez-Hernández et al. revealed clear socioeconomic inequalities among elderly Spanish cardiovascular patients, particularly linked to education and occupation [18]. Similarly, Haeberer et al. found disparities in ischemic heart disease and heart failure that were gender based, with women being at greater risk; however, they reported no gender-based inequalities in cerebrovascular diseases [19]. Although cardiovascular complications among T2DM patients are well-documented in Saudi Arabia, there remains a notable gap in research focusing on socioeconomic inequality as a risk factor. Therefore, this study aims to investigate the association between social inequalities and cardiovascular complications among patients with T2DM, addressing a gap in regional evidence. This study specifically examines macrovascular complications, which are distinct from microvascular outcomes previously explored in related research using the same cohort.

In this study, socioeconomic disparities among patients with type 2 diabetes mellitus (T2DM) were analyzed in relation to cardiovascular complications. The results indicated elevated odds of hypertension among specific groups: divorced or widowed individuals, those with lower educational attainment, individuals with higher income, patients with poor glycemic control and longer duration of diabetes, those who were retired or unemployed, and residents living outside of Riyadh. These associations may reflect a range of underlying factors, including limited social support for divorced or widowed individuals, inadequate knowledge of diabetes self-management among less educated patients, sedentary behavior and low adherence to treatment among those with T2DM, progression of disease complications, age-related frailty, and reduced access to healthcare services.

These findings are consistent with the study by Tao et al., which demonstrated that lower socioeconomic status significantly correlates with increased risk of T2DM complications, including poor blood pressure control and hypertension [30]. Furthermore, Fano et al. also reported that females with T2DM are more likely to experience hypertension and other diabetes-related complications [31]. However, a point of divergence in this study relates to income, as Fano et al. found that lower income was associated with higher risk, whereas this study observed higher odds of complications among higher income groups [31].

Although the raw prevalence of dyslipidemia was highest in the lowest income group, after adjusting for other factors, higher-income individuals exhibited greater odds of having dyslipidemia. One explanation is that higher income may be linked to greater access to unhealthy dietary choices, which could worsen lipid profiles. However, this paradox could also be partly explained by differences in healthcare access and better diagnostic opportunities among high-income groups, which may lead to more frequent testing and earlier detection of dyslipidemia.

These findings are consistent with the study by Al-Hassan et al., which identified a higher prevalence of dyslipidemia among female patients compared to their male counterparts [32]. In contrast, Al-Nozha et al. reported that males were at greater risk of dyslipidemia than females, highlighting a discrepancy in gender-related findings [33]. The current results are also in alignment with those of Al-Kaabba et al., who found significant associations between dyslipidemia and sociodemographic factors such as lower education, marital status, and unemployment or retirement [34]. However, differences emerged in this study regarding the role of gender and income, where high income—unlike in some prior research—was linked to increased DLD risk.

Furthermore, the study’s findings echo those of Tao et al. and Fano et al., both of whom reported that socioeconomic factors including low education, female gender, and poor glycemic control significantly contribute to the prevalence of dyslipidemia among T2DM populations [30,31].

The results of this study indicated that the likelihood of experiencing stroke was significantly higher among female patients, those who were divorced or separated, individuals with lower educational levels, middle-income earners, patients with poor glycemic control, individuals who had lived with diabetes for over 15 years, those who were retired or unemployed, and those residing outside the Riyadh region. Similarly, the risk of acute coronary syndrome (ACS) was notably greater among females, divorced or widowed individuals, patients with university-level education, those in the middle-income bracket, individuals with poorly controlled diabetes, patients with a diabetes duration of 6 to 15 years, and those who were not working or lived outside Riyadh.

These findings are supported by Memon et al., who reported that females had a higher risk of developing ACS [35], and by Wong et al., who emphasized that marital status plays a significant role in predicting cardiovascular outcomes, including ACS [36]. One plausible explanation for these associations is the absence of adequate social support, which can negatively impact patients’ ability to adhere to self-care behaviors and manage their condition effectively. This aligns with findings by Wiesmaierova et al., who observed that partner support had a protective effect, mitigating adverse outcomes among patients with cardiac conditions [37].

Additionally, the results are consistent with the study by Shrivastava et al., who found that women with low socioeconomic status were more vulnerable to both stroke and ACS. This vulnerability was largely attributed to limited healthcare access and inadequate resources in rural areas, contributing to low levels of self-management among chronically ill diabetic patients in India [38].

The differing risk patterns between ACS and stroke concerning educational attainment may be due to the relative complexity of these conditions. ACS, as a more complex clinical syndrome, may require a higher level of health literacy and disease awareness, which could explain its stronger association with educational background.

This study had several limitations. First, it was cross-sectional in nature, limiting causal inference. Second, the COVID-19 pandemic imposed significant constraints, including challenges in accessing certain patient records and potential selection bias. Third, diabetic nephropathy was estimated solely based on eGFR, without incorporating microalbuminuria data, which may have led to an underestimation of early kidney damage. Lastly, some variables relied on self-reported data, which could be subject to recall bias.

## 5. Conclusions

This study identified notable social disparities among patients with type 2 diabetes mellitus (T2DM) who also suffer from cardiovascular conditions, specifically stroke, dyslipidemia (DLD), and acute coronary syndrome (ACS). These inequalities were significantly associated with various sociodemographic and clinical factors, including marital status, educational attainment, glycemic control, income level, duration of diabetes, and employment status. In light of these findings, the study recommends enhancing healthcare accessibility for patients residing outside the central hospital areas, ensuring equitable health services for populations regardless of their geographic location within or beyond the Riyadh region. Furthermore, it advocates for targeted awareness campaigns tailored to individuals with diabetes, aiming to improve their understanding and application of self-care practices. Such interventions could play a crucial role in mitigating complications and enhancing health outcomes for this high-risk population.

## Figures and Tables

**Table 1:** Sociodemographic characteristics.

Variable	M ± SD	F(%)
Age	57.93 ± 10.20	
1. Gender		
2. Female		287 (50)
3. Male		287 (50)
Marital Status		
1. Single		10 (1.7)
2. Married		460 (80.1)
3. Divorced		31 (5.4)
4. Widowed		73 (12.7)
Educational level		
1. Illiterate		117 (20.4)
2. Primary school		100 (17.4)
3. Intermediate school		73 (12.7)
4. Tertiary school		114 (19.9)
5. University degree		170 (29.6)
Nationality		
1. Saudi		535 (93.2)
2. Non-Saudi		39 (6.8)
Main Work		
1. Working		160 (27.9)
2. Not working (able to work)		12 (2.1)
3. Not working (unable to work)		10 (1.7)
4. Homemaker (housewife)		212 (36.9)
5. Retired		180 (31.4)
Monthly income		
1. ≤3000 SAR		148 (25.8)
2. 3001–6000		123 (21.4)
3. 6001–9000		91 (15.9)
4. 9001–12,000		51 (8.9)
5. ≥12,000		161 (28)
Living region		
1. Inside Riyadh		485 (84.5)
2. Outside Riyadh		89 (15.5)
Housing Unit		
1. Traditional house		35 (6.1)
2. A floor in a villa		35 (6.1)
3. A floor in a traditional house		12 (2.1)
4. Villa		408 (71.1)
5. Apartment		81 (14.1)
6. Other		3 (5)
Duration of DM (years)	14.74 ± 8.9	
How frequent are you following up for diabetes?		
1. Monthly		15 (2.6)
2. Every 2 months		6 (1.0)
3. Every 3 months		336 (58.5)
4. Every 6 months		186 (32.4)
5. Every 1 year		18 (3.1)
6. Irregular		13 (2.3)
Where do you usually follow-up for your diabetes?		
1. Primary healthcare center		9 (1.6)
2. Diabetic center		79 (13.8)
3. Public hospital		475 (82.8)
4. Private centers		11 (1.9)
Type of management		
1. Diet only		5(0.9)
2. Oral tablets		231 (40.2)
3. Insulin		36 (6.3)
4. Oral and insulin		302 (52.6)

**Table 2:** Multivariate logistic regression analysis for SES and hypertension.

	n	HTN (n)	Prevalence HTN (%)	Model 1		*p* Value	Model 2		*p* Value	Model 3		*p* Value
				OR	95% CI		OR	95% CI		OR	95% CI	
**Gender**												
Male	287	183	63.8	1.00			1.00			1.00		
Female	287	195	67.9	2.13	(0.61–2.33)	0.11	2.13	(0.51–2.21)	0.09	2.29	(0.57–2.34)	0.08
**Marital status**												
Single	10	4	40	1.00			1.00			1.00		
Married	460	293	63.7	1.88	(0.66–2.44)	0.02	2.11	(0.97–2.26)	0.01	2.33	(0.59–3.47)	0.01
Divorced or widowed	104	81	77.8	1.67	(1.13–1.98)		1.88	(1.47–2.56)		2.36	(2.07–4.22)	
**Educational level**						0.07			0.06			0.04
Illiterate	117	95	81.2	1.00			1.00			1.00		
Secondary school or less	287	186	64.8	2.07	(0.12–3.03)		2.57	(1.18–3.26)		2.64	(0.67–4.24)	
University education	170	97	57.1	1.09	(0.37–1.39)		1.33	(0.48–2.37)		1.46	(1.27–3.48)	
**Monthly income**						0.04			0.03			0.02
≤3000 SAR	148	109	73.6	1.00			1.00			1.00		
3001–6000	214	140	65.4	2.25	(0.78–3.93)		2.18	(1.16–4.83)		2.44	(2.23–4.18)	
More than 6000 SAR	212	129	60.8	1.18	(1.03–2.09)		2.07	(2.07–6.15)		2.49	(0.81–3.29)	
**Most recent fasting blood sugar measurement (mg/dL)**						0.03			0.02			0.01
Normal	182	112	61.5	1.00			1.00		0.04	1.00		0.03
Poor glycemic control	392	266	67.9	2.11	(1.97–2.77)		2.46	(2.14–3.37)		2.51	(2.09–3.59)	
**Duration of diabetes**												
Less than 6 years	119	53	44.5	1.00			1.00			1.00		
6–15 years	218	139	63.8	2.41	(0.63–3.67)	0.05	2.33	(1.03–3.84)	0.04	2.53	(1.10–3.74)	0.03
More than 15 years	237	186	78.5	1.64	(0.51–2.69)		1.39	(0.68–1.97)		2.57	(1.28–3.14)	
**Main Work**						0.03			0.02			
Working	160	88	55	1.00			1.00			1.00		
Retired or not working	202	140	69.3	2.19	(1.64–4.37)		2.91	(2.21–6.14)		3.18	(2.16–4.06)	0.01
Housewife or homemaker	212	150	70.8	2.27	(2.54–3.48)		2.54	(1.19–3.38)		2.69	(1.70–3.82)	
**Living region**						0.12			0.13			0.14
Inside Riyadh	485	323	66.6	1.00			1.00			1.00		
Outside Riyadh	89	55	61.8	1.91	(0.49–2.09)		2.04	(0.31–3.51)		2.17	(0.37–2.88)	

Model 1: Adjusted for age; Model 2: Model 1 + sex, marital status, educational level, monthly income, and main work; Model 3: Model two + duration of diabetes + most recent fasting blood sugar measurement (mg/dL).

**Table 3:** Multivariate logistic regression analysis for SES and DLD.

	n	DLD(n)	Prevalence DLD (%)	Model 1			Model 2			Model 3		*p* Value
				OR	95% CI	*p* Value	OR	95% CI	*p* Value	OR	95% CI	
**Gender**												
Male	287	225	78.4	1.00			1.00			1.00		
Female	287	230	80.1	2.16	(1.17–3.21)	0.03	2.32	(0.49–2.37)	0.07	2.59	(1.18–2.87)	0.04
**Marital status**												
Single	10	6	60	1.00			1.00			1.00		
Married	460	365	79.3	2.49	(1.18–3.89)	0.01	2.58	(1.51–3.74)	0.02	3.01	(1.87–3.08)	0.01
Divorced or widowed	104	84	80.8	2.48	(1.55–2.41)		2.74	(1.33–2.97)		2.88	(1.76–3.45)	
**Educational level**												0.02
Illiterate	117	99	84.6	1.00			1.00			1.00		
Secondary school or less	287	227	79.1	1.16	(0.13–2.57)	0.11	2.08	(2.20–3.15)	0.03	2.22	(2.01–3.33)	0.02
University education	170	129	75.9	1.23	(0.26–1.58)		1.16	(1.21–2.57)		1.25	(0.55–1.63)	
**Monthly income**					0.02				0.03			0.01
≤3000 SAR	148	122	82.4	1.00			1.00			1.00		
3001–6000	214	168	78.5	2.09	(2.51–3.48)		2.18	(4.15–4.99)		2.41	(2.33–3.01)	
More than 6000 SAR	212	165	77.8	1.88	(1.54–2.34)		2.27	(0.51–2.38)		2.59	(1.63–2.70)	
**Most recent fasting blood sugar measurement (mg/dL)**						0.04			0.04			0.02
Normal	182	138	75.8	1.00			1.00			1.00		
Poor glycemic control	392	317	80.9	1.38	(1.05–2.41)		1.79	(1.64–2.15)		2.09	(1.75–2.29)	
**Duration of diabetes**						0.12			0.05			0.01
Less than 6 years	119	79	66.4	1.00			1.00			1.00		
6–15 years	218	169	77.5	1.05	(1.71–2.25)		2.17	(1.88–3.24)		2.28	(2.02–3.16)	
More than 15 years	237	207	87.3	2.11	(0.33–2.64)		2.41	(0.71–2.68)		2.86	(1.81–4.63)	
**Main Work**						0.03			0.06			0.04
Working	160	116	72.5	1.00			1.00			1.00		
Retired or not working	202	162	80.2	2.33	(1.28–2.57)		2.49	(1.61–3.52)		2.96	(2.29–4.33)	
Housewife or homemaker	212	177	83.5	1.87	(0.36–3.51)		2.07	(1.42–4.69)		2.61	(1.80–4.06)	
**Living region**						0.11			0.05		0.03	
Inside Riyadh	485	381	78.6	1.00			1.00			1.00		
Outside Riyadh	89	74	83.1	1.32	(0.55–2.25)		1.41	(0.93–2.37)		2.18	(0.31–2.51)	

Model 1: Adjusted for age; Model 2: Model 1 + sex, marital status, educational level, monthly income, and main work; Model 3: Model two + duration of diabetes + most recent fasting blood sugar measurement (mg/dL).

**Table 4:** Multivariate logistic regression analysis for SES and ACS.

	n	ACS (n)	Prevalence ACS (%)	Model 1			Model 2			Model 3		
				OR	95% CI	*p* Value	OR	95% CI	*p* Value	OR	95% CI	*p* Value
**Gender**												
Male	287	40	13.9	1.00			1.00			1.00		
Female	287	22	7.7	1.77	(0.67–2.41)	0.03	2.07	(1.65–2.12)	0.07	2.35	(2.13–3.01)	0.01
**Marital status**												
Single	10	0	0	1.00			1.00			1.00		
Married	460	49	10.7	2.06	(0.79–3.33)	0.01	2.15	(0.62–2.54)	0.02	2.39	(2.18–2.57)	0.04
Divorced or widowed	104	10	9.6	2.09	(1.41–2.56)		2.23	(1.79–3.16)		2.91	(1.62–4.07)	
**Educational level**						0.11			0.03			0.01
Illiterate	117	12	10.3	1.00			1.00			1.00		
Secondary school or less	287	36	12.5	1.88	(0.33–2.57)		2.16	(0.58–2.54)		2.34	(0.54–2.91)	
University education	170	14	8.2	1.12	(0.31–3.59)		1.49	(1.21–3.17)		2.64	(2.00–3.67)	
**Monthly income**						0.01			0.01			0.02
≤3000 SAR	148	13	8.8	1.00			1.00			1.00		
3001–6000	214	24	11.2	2.19	(1.41–3.99)		2.35	(1.08–4.13)		2.61	(2.51–4.16)	
More than 6000 SAR	212	25	11.8	2.27	(1.15–3.62)		2.41	(1.08–2.87)		2.14	(1.29–2.76)	
**Most recent fasting blood sugar measurement (mg/dL)**						0.01			0.01			0.01
Normal	182	16	8.8	1.00	0.01		1.00		0.01	1.00		0.02
Poor glycemic control	392	46	11.7	1.16	(1.07–1.45)		1.64	(1.37–2.13)		1.81	(1.69–3.05)	
**Duration of diabetes**						0.12			0.04			0.14
Less than 6 years	119	3	2.5	1.00			1.00			1.00		
6–15 years	218	20	9.1	1.99	(0.86–3.12)		2.11	(1.84–2.53)		2.38	(2.17–3.43)	
More than 15 years	237	39	16.5	1.96	(1.91–2.15)		1.72	(1.07–3.15)		2.09	(0.59–2.63)	
**Main Work**						0.02			0.03			0.03
Working	160	16	10	1.00			1.00			1.00		
Retired or not working	202	31	15.3	1.96	(1.31–2.17)		2.26	(1.49–3.51)		2.79	(2.36–3.17)	
Housewife or homemaker	212	15	7.1	1.14	(0.79–2.88)		1.21	(1.08–2.28)		1.30	(0.59–2.25)	
**Living region**						0.14			0.13			0.14
Inside Riyadh	485	54	11.1	1.00			1.00			1.00		
Outside Riyadh	89	8	9	1.37	(0.38–1.52)		1.40	(0.89–2.23)		1.66	(1.03–3.14)	

Model 1: Adjusted for age; Model 2: Model 1 + sex, marital status, educational level, monthly income, and main work; Model 3: Model two + duration of diabetes + most recent fasting blood sugar measurement (mg/dL).

**Table 5:** Multivariate logistic regression analysis for SES and stroke.

	n	Stroke (n)	Prevalence Stroke (%)	Model 1			Model 2		*p* Value	Model 3		*p* Value
				OR	95% CI	*p* Value	OR	95% CI		OR	95% CI	
**Gender**												
Male	287	22	7.7	1.00			1.00			1.00		
Female	287	12	4.2	1.75	(1.13–2.56)	0.04	1.83	(0.27–2.68)	0.06	2.17	(2.12–2.59)	0.03
**Marital status**												
Single	10	0	0	1.00			1.00			1.00		
Married	460	24	5.2	2.13	(1.51–3.03)	0.02	2.25	(2.16–3.39)	0.01	2.41	(1.17–3.84)	0.01
Divorced or widowed	104	10	9.6	2.51	(1.51–2.70)		2.69	(2.41–3.55)		2.83	(2.30–3.57)	
**Educational level**						0.05			0.04			0.02
Illiterate	117	9	7.7	1.00			1.00			1.00		
Secondary school or less	287	15	5.2	2.19	(1.05–2.89)		2.36	(2.07–3.61)		2.88	(1.01–5.15)	
University education	170	10	5.9	1.96	(0.25–2.18)	0.12	2.14	(2.10–3.57)	0.05	2.20	(0.71–2.67)	0.04
**Monthly income**												
≤3000 SAR	148	14	9.5	1.00			1.00			1.00		
3001–6000	214	9	4.2	1.84	(0.98–3.16)	0.06	2.15	(2.51–3.66)	0.04	2.64	(1.69–3.08)	0.03
More than 6000 SAR	212	11	5.2	1.05	(1.51–5.07)		2.24	(2.18–3.55)		2.51	(1.15–3.61)	
**Most recent fasting blood sugar measurement (mg/dL)**												
Normal	182	10	5.5	1.00			1.00			1.00		
Poor glycemic control	392	24	6.1	1.36	(1.17–1.48)	0.09	1.28	(1.18–1.74)	0.08	1.41	(1.32–1.79)	0.01
**Duration of diabetes**												
Less than 6 years	119	2	1.7	1.00			1.00			1.00		
6–15 years	218	9	4.1	1.84	(0.77–3.18)		1.97	(0.21–2.67)		2.19	(1.41–5.18)	
More than 15 years	237	23	9.7	2.17	(1.29–3.12)	0.02	2.39	(1.18–2.69)	0.13	2.89	(1.17–5.66)	0.15
**Main Work**												
Working	160	6	1	1.00			1.00			1.00		
Retired or not working	202	38	18.8	2.61	(1.42–2.88)	0.03	2.83	(1.27–5.12)	0.04	3.18	(2.94–3.67)	0.04
Housewife or homemaker	212	11	5.2	2.16	(1.18–4.51)		2.27	(1.18–2.66)		2.41	(0.71–2.48)	
**Living region**												
Inside Riyadh	485	26	5.4	1.00			1.00			1.00		
Outside Riyadh	89	8	9	1.35	(1.11–3.48)	0.01	1.34	(0.58–2.69)	0.03	1.52	(0.81–3.00)	0.01

Model 1: Adjusted for age; Model 2: Model 1 + sex, marital status, educational level, monthly income, and main work; Model 3: Model two + duration of diabetes + most recent fasting blood sugar measurement (mg/dL).

## Data Availability

The data presented in this study are available on request from the corresponding author.
